# Crystal clear: visualizing the intervention mechanism of the PD-1/PD-L1 interaction by two cancer therapeutic monoclonal antibodies

**DOI:** 10.1007/s13238-016-0337-7

**Published:** 2016-11-04

**Authors:** Shuguang Tan, Danqing Chen, Kefang Liu, Mengnan He, Hao Song, Yi Shi, Jun Liu, Catherine W.-H. Zhang, Jianxun Qi, Jinghua Yan, Shan Gao, George F. Gao

**Affiliations:** 1 CAS Key Laboratory of Pathogenic Microbiology and Immunology, Institute of Microbiology, Chinese Academy of Sciences, Beijing, 100101 China; 2 National Institute for Viral Disease Control and Prevention, Chinese Center for Disease Control and Prevention (China CDC), Beijing, 102206 China; 3 College of Laboratory Medicine and Life Sciences, Wenzhou Medical University, Wenzhou, 325035 China; 4 College of Life Sciences, University of Chinese Academy of Sciences, Beijing, 100049 China; 5 Research Network of Immunity and Health (RNIH), Beijing Institutes of Life Science, Chinese Academy of Sciences, Beijing, 100101 China; 6 ImmuFucell Biotechnology Co.Ltd., Beijing, 100102 China; 7 CAS Key Laboratory of Microbial Physiological and Metabolic Engineering, Institute of Microbiology, Chinese Academy of Sciences, Beijing, 100101 China; 8 CAS Key Laboratory of Bio-medical Diagnostics, Suzhou Institute of Biomedical Engineering and Technology, Chinese Academy of Sciences, Suzhou, Jiangsu 215163 China; 9 Savaid Medical School, University of Chinese Academy of Sciences, Beijing, 100049 China

**Keywords:** PD-1/PD-L1 interaction, checkpoint blockade, molecular basis, therapeutic antibody

## Abstract

Antibody-based PD-1/PD-L1 blockade therapies have taken center stage in immunotherapies for cancer, with multiple clinical successes. PD-1 signaling plays pivotal roles in tumor-driven T-cell dysfunction. In contrast to prior approaches to generate or boost tumor-specific T-cell responses, antibody-based PD-1/PD-L1 blockade targets tumor-induced T-cell defects and restores pre-existing T-cell function to modulate antitumor immunity. In this review, the fundamental knowledge on the expression regulations and inhibitory functions of PD-1 and the present understanding of antibody-based PD-1/PD-L1 blockade therapies are briefly summarized. We then focus on the recent breakthrough work concerning the structural basis of the PD-1/PD-Ls interaction and how therapeutic antibodies, pembrolizumab targeting PD-1 and avelumab targeting PD-L1, compete with the binding of PD-1/PD-L1 to interrupt the PD-1/PD-L1 interaction. We believe that this structural information will benefit the design and improvement of therapeutic antibodies targeting PD-1 signaling.

## INTRODUCTION

The host immune system is critical for defending against microbial pathogens and “non-self” malignant cells to maintain health. T-cell immune responses play pivotal roles in adoptive immune responses by directly killing target cells or indirect modulation via cytokines (Palucka and Coussens, 2016). Naïve T-cell activation involves both T-cell receptor (TCR)/peptide major histocompatibility complex (pMHC) interactions and co-stimulatory ligand-receptor interactions, the two-signal model proposed by Lafferty and Cunningham (Bretscher and Cohn, 1970; Lafferty and Cunningham, 1975; Cunningham and Lafferty, 1977; Gao and Jakobsen, 2000; Gao et al., 2002). Additionally, activated T cells also require co-stimulatory and co-inhibitory molecules to modulate TCR-mediated T-cell responses and self tolerance (Gao and Jakobsen, 2000; Gao et al., 2002). The most important co-stimulatory and co-inhibitory molecules involve B7-CD28 superfamily- and TNF-TNF receptor superfamily-related ligands and receptors. Programmed cell death 1 (PD-1) is a member of the CD28 superfamily and was first discovered as a gene upregulated in a T cell hybridoma undergoing cell death (Ishida et al., 1992). The negative regulatory function of PD-1 in T-cell activation was revealed in *Pdcd1−/−* mice that are genetically predisposed to systematic autoimmunity (Nishimura et al., 1999). PD-1 ligand 1 (PD-L1) and PD-1 ligand 2 (PD-L2) were identified to be the ligands (PD-Ls) of PD-1 in 2000 and 2001, respectively (Freeman et al., 2000; Latchman et al., 2001a, b; Tseng et al., 2001). Subsequently, exhausted T-cell function reversion was achieved through the blockade of the PD-1/PD-L1 interaction with antibodies that restored the exhausted CD8^+^ T-cell reactivity and regained their antitumor activity (Curiel et al., 2003; Hirano et al., 2005). Moreover, PD-1/PD-L1 signaling is important in the maintenance of T-cell exhaustion during chronic viral infection, and antibody blockade of the PD-1/PD-L1 interaction restores function in exhausted CD8^+^ T cells (Barber et al., 2006a). Other well-known co-inhibitory and co-stimulatory molecules include CTLA-4, LAG-3, CD226-TIGIT-CD96, TIM, and the TNF-TNF receptor (*e.g.*,4-1BB, OX-40, and GITR) families, etc. (Schildberg et al., 2016). Because T-cell activation or exhaustion depends strongly on the co-stimulatory and co-inhibitory signaling pathways, co-stimulatory and co-inhibitory molecules are also called “immune checkpoint” molecules (Tan and Gao, 2015; Callahan et al., 2016).

The breakthrough of antibody-based checkpoint blockade in cancer treatment in the last few years has given rise to a promising future for cancer immunotherapies (Callahan et al., 2016). Checkpoint blockade takes advantage of a monoclonal antibody (MAb) that blocks co-inhibitory signaling pathways to restore T-cell function (Barber et al., 2006b; John et al., 2013). Multiple PD-1/PD-L1 blockade antibodies have been approved for clinical use or have entered into clinical trials, such as pembrolizumab, nivolumab, and atezolizumab, and have shown great efficacies to treat multiple advanced-stage tumors (Powles et al., 2014; Chapman et al., 2015; Postow et al., 2015; Robert et al., 2015b). Previously, the molecular basis of PD-1/PD-L1 blockade and tumor immunotherapy has been thoroughly reviewed (Chen and Han, 2015; Li et al., 2016; Zou et al., 2016), we briefly overviewed the current understanding of the molecular mechanisms of the PD-1/PD-L1 interaction and focused on the recently defined structural basis of the therapeutic antibody-based PD-1/PD-L1 blockade in the present review.

## EXPRESSION AND INHIBITORY FUNCTIONS OF PD-1/PD-Ls

### Tissue tropism of PD-1 and PD-L1/L2 expression and regulation

As a co-inhibitory molecule of the B7/CD28 family, PD-1 negatively regulates T-cell responses to both internal and external antigens upon binding to its ligands PD-L1 or PD-L2 (Callahan et al., 2016). Inducible expression of PD-1 is observed in T and B lymphocytes, dendritic cells (DCs), natural killer cells, monocytes, and macrophages during immune activation and chronic inflammation (Nishimura et al., 1996; Petrovas et al., 2006; Chang et al., 2008; Liu et al., 2009). On T cells, PD-1 can be induced following TCR-mediated activation and/or cytokine stimulation (Agata et al., 1996; Kinter et al., 2008). The elevated PD-1 levels progressively render antigen-specific T cells susceptible to exhaustion or anergy during chronic infections or tumor development (Blank et al., 2006; Blackburn et al., 2009). Aside from immune cells, PD-1 expression has also been detected in tumor cells. Indeed, melanoma cell-intrinsic PD-1 promotes tumorigenesis by modulating downstream mTOR signaling (Kleffel et al., 2015).

The two PD-1 ligands also show distinct expression patterns. PD-L1 is widely expressed in a variety of hematopoietic and non-hematopoietic cells, while PD-L2 expression is restricted to antigen-presenting cells, macrophages, T helper 2 cells, and non-hematopoietic cells in the lung (Dong et al., 2002; Yamazaki et al., 2002; Ohigashi et al., 2005; Hamanishi et al., 2007; Nomi et al., 2007; Lesterhuis et al., 2011). Elevated PD-L1 expression on multiple tumor cells is also an important mechanism of tumor-induced immune escape (Iwai et al., 2002; Kataoka et al., 2016).

### PD-1 signaling and PD-1-induced T-cell exhaustion

T-cell exhaustion is defined as dysfunction of T cells during chronic virus infection or cancer (Curiel et al., 2003; Barber et al., 2006b). Progressive loss of T-cell function occurs in a hierarchical manner, where T cells lose the distinct properties of IL-2 production and the ability to proliferate at the first step and then fail to produce TNF-α and IFN-γ at later stages (Wherry et al., 2003). The PD-1 pathway serves as a critical regulator of T-cell exhaustion state (Freeman et al., 2000). The cytoplasmic domain of PD-1 contains an immunoreceptor tyrosine-based inhibition motif (ITIM) and an immunoreceptor tyrosine-based switch motif (ITSM). Both of these motifs contribute to PD-1-mediated T-cell inhibition (Chatterjee et al., 2013). Binding of the PD-L1 or PD-L2 to PD-1 induces phosphorylation on ITIM (Y223) and ITSM (Y248) tyrosine residues, thus leading to recruitment of Src homology region 2 domain-containing protein tyrosine phosphatases (SHP-1 and SHP-2) and subsequent down regulation of TCR signaling through dephosphorylation of signaling intermediates such as CD3ζ, ZAP70, and PKCθ in T cells (Okazaki et al., 2001; Chemnitz et al., 2004; Sheppard et al., 2004). However, it is unclear how the cytoplasmic motif recruits intracellular factors and how the cytoplasmic domain interacts with these factors.

### PD-1 and PD-L1 upregulation in the tumor microenvironment and tumor-induced immunosuppression

Studies show that co-inhibitory molecules such as PD-1 and PD-L1 induce immune suppression in the tumor microenvironment (Iwai et al., 2002; Blank et al., 2006; Blackburn et al., 2009; Kataoka et al., 2016). To date, expression of PD-L1 is detected in multiple solid tumors, including melanoma, lung, breast, and ovarian cancers, as well as in myeloma, T cell lymphoma, *etc*. (Brown et al., 2003; Wherry et al., 2003; Ghebeh et al., 2006; Hamanishi et al., 2007; Liu et al., 2007; Hino et al., 2010). Moreover, PD-L1 expression can be detected in myeloid DCs, which is induced by factors in the tumor microenvironment (Curiel et al., 2003). The PD-L1 expression levels on tumor cells tend to be associated with tumor progression and are predictive of unfavorable prognosis and better response to PD-1 blockade treatment, to a certain extent, in ovarian, kidney, pancreatic, and gastric cancers (Thompson et al., 2005; Wu et al., 2006; Hamanishi et al., 2007; Nomi et al., 2007; Garon et al., 2015; Gandini et al., 2016). PD-1 expressed by T lymphocytes, particularly tumor-infiltrating lymphocytes (TILs), can lead to dysfunction of tumor-specific T cells to eliminate tumors (Tumeh et al., 2014). Elevated expression of PD-1 on CD4^+^ T cells in Hodgkin lymphoma negatively affects CD4^+^ T cells and is suspected to facilitate immune evasion of the tumor cells (Chemnitz et al., 2007). Elevated expression of PD-1 is also observed in CD4^+^ T cells rather than CD8^+^ T cells in adult T-cell leukemia/lymphoma (Shimauchi et al., 2007).

## ANTIBODY-BASED PD-1/PD-L1 IMMUNE CHECKPOINT BLOCKADE FOR TUMOR THERAPY

### The mechanism of PD-1/PD-L1 interaction interference for reactivating immune activity

Forced expression of PD-1 and PD-L1 by T cells and tumor cells underlies the rationale that blockade of the PD-1 pathway would restore tumor-specific T-cell function to eliminate tumor cells (Curiel et al., 2003). Targeting the PD-1 pathway may induce T-cell immune responses via the followings: 1) Activation of T cells. The PD-1/PD-L1 interaction would block the TCR-driven “stop signal” that limits T-cell mobility and thereby interrupts T cell-DC contacts and T-cell activation, proliferation, and cytokine production (Benvenuti et al., 2004). Antibodies that block PD-1/PD-L1 interaction would result in alteration of T-cell motility and promotion of T cell-DC contacts. 2) Diminishment of T-cell exhaustion. Persistent PD-1 expression could result in T-cell exhaustion, which is reversible by blocking the PD-1 pathway. Upregulation of PD-1 on CD8^+^ T cells in the tumor microenviroment is suggested to reflect exhaustion or anergy of T cells accompanied by the reduction of cytokine production (Ahmadzadeh et al., 2009). 3) Inhibition of Treg cells. There is a recent report that PD-1 play critical roles in modulating the activation threshold and maintaining the balance between regulatory and effector T cells (Zhang et al., 2016). Further, infiltration of PD-1-positive Treg cells into tumors can hinder the proliferation and function of effector CD8^+^ T cells (Wang et al., 2004; Francisco et al., 2009). In summary, blockade of the PD-1 pathway can effectively induce anti-tumor immune responses by restoration of T-cell function and inhibiting intratumoral Treg cells within the tumor microenvironment.

It is noting that PD-L1 also interacts with CD80 to inhibit T cells, while PD-L2 binds to repulsive guidance molecule b (RGMb) to mediate respiratory tolerance (Butte et al., 2007; Xiao et al., 2014). Antibodies targeting PD-1 would block PD-1/PD-L1 or PD-1/PD-L2 interactions, leaving PD-L1/CD80 and PD-L2/RGMb signaling unaffected. On the other hand, though PD-1/PD-L1 signal would be blocked by PD-L1 targeted MAbs, the PD-1/PD-L2 interaction would not be abrogated during administration of anti-PD-L1 antibodies. Additionally, other inhibitory molecules also play important roles with similar or distinct inhibitory pathways compared to the PD-1 pathway. Combination therapies with different checkpoint blockade agents might improve tumor regression efficiency, and multiple combination therapies involving different checkpoint blockade agents are now in clinical trials (Mahoney et al., 2015).

### Clinical findings of PD-1/PD-L1 immune checkpoint blockade therapy

The US Food and Drug Administration (FDA) has approved two PD-1-targeted MAbs, nivolumab from Bristol-Myers Squibb (Opdivo, also known as BMS-936558, MDX-1106, and ONO-4538) and pembrolizumab from Merck (Keytruda, also known as lambrolizumab and MK-3475), for advanced melanoma, non-small cell lung cancer (NSCLC), and kidney cancer. In 2016, the US FDA gave accelerated approval to atezolizumab from Genentech (Tecentriq, also known as MPDL-3280A) for the treatment of patients with locally advanced or metastatic urothelial carcinoma. Further, various MAbs targeting the PD-1 pathway are being developed and evaluated in numorous clinical trials involving thousands of patients (Table 1). Most of the PD-1-targeted therapeutic antibodies are IgG4 human or humanized MAbs that block the PD-1/PD-L1 or PD-1/PD-L2 interaction to restore tumor-specific T cell reactivity without mediating antibody-dependent cell-mediated cytotoxicity (ADCC). PD-L1-targeted therapeutic antibodies possess PD-1/PD-L1 blockade activity with or without ADCC activity.

**Table 1:** PD-1- and PD-L1-blocking antibodies under clinical development

Target	Agent^a^	NCT number^b^	Targeted diseases	Antibody class	Developer	Stage of development
PD-1	Nivolumab (BMS-936558/MDX-1106/ONO-4538)	NCT01658878, NCT01844505, NCT02596035, NCT02017717, NCT02105636, *etc.*	Non-small cell lung cancer (NSCLC), melanoma, renal cell carcinoma, colon cancer, glioblastoma, head and neck carcinoma, hepatocellular carcinoma, *etc.*	Human IgG4	Bristol-Myers Squibb	FDA approved (melanoma, NSCLC, kidney cancer)
	Pembrolizumab (MK-3475)	NCT02834052, NCT01295827, NCT02444741, NCT02819518, NCT02231749, *etc.*	NSCLC, triple negative breast cancer, renal cell carcinoma, melanoma, colon cancer, *etc.*	Humanized IgG4	Merck & Co., Inc., USA	FDA approved (melanoma, NSCLC)
	MEDI0680 (AMP-514)	NCT02118337, NCT02013804, NCT02271945	Advanced malignancies, relapsed/refractory aggressive B-cell lymphomas	Humanized IgG4	Medimmune	Phase I/II
	REGN2810	NCT02760498, NCT02383212, NCT02520245	Advanced cutaneous squamous cell carcinoma, advanced malignancies	Human IgG4	Regeneron/Sanofi	Phase I/II
	PDR001	NCT02795429, NCT02829723, NCT02404441, NCT02740270, NCT02605967, *etc.*	Advanced hepatocellular carcinoma, melanoma, NSCLC, triple negative breast cancer,lymphomas, nasopharyngeal carcinoma, *etc.*	Humanized IgG4	Novartis	Phase I/II
	BGB-A317	NCT02407990, NCT02660034, NCT02795182	Advanced tumors, lymphoma, leukemia	Humanized IgG4	BeiGene	Phase I
	Pidilizumab (CT-011, MDV9300)	NCT01096602, NCT02530125, NCT01420965, NCT01441765, NCT01067287, NCT01313416, *etc.*	Acute myelogenous leukemia, stage III-IV diffuse large B-cell lymphoma, prostatic neoplasms, renal cell carcinoma, multiple myeloma, pancreatic cancer, *etc.*	Humanized IgG1	Medivation	Phase II
	Shr 1210	NCT02492789, NCT02738489, NCT02721589, NCT02742935	Melanoma, neoplasm, lung cancer, breast cancer	Human IgG4	Incyte/Jiangsu HengRui	Phase I
	Js001	NCT02836795, NCT02836834, NCT02838823	Melanoma, urological cancer, lymphoma, lung cancer, breast cancer	Humanized mab	Shanghai Junshi Bioscience	Phase I
	Tsr-042	NCT02715284	Advanced or metastatic solid tumor	Humanized mab	Tesaro	Phase I
PD-L1	Atezolizumab (MPDL-3280A)	NCT02657434, NCT02420821, NCT02425891, *etc.*	NSCLC, renal cell carcinoma, triple negative breast cancer, *etc.*	Humanized IgG1	Genentech /Roche	FDA approved (urothelial carcinoma)
	Durvalumab (MEDI4736)	NCT02516241, NCT02454933, NCT02369874, NCT02125461, *etc.*	NSCLC, bladder cancer, head and neck cancer, EGFR T790M^+^ NSCLC, triple negative breast cancer, *etc.*	Human IgG1	Medimmune/Astrazeneca	Phase III
	Avelumab (MSB0010718C)	NCT02603432, NCT02718417, NCT02395172, NCT02625610, *etc.*	Gastric cancer, urothelial cancer, ovarian cancer, NSCLC, *etc.*	Human IgG1	Merck Serono/ Pfizer	Phase III
	BMS-936559 (MDX-1105)	NCT02576457, NCT02028403, NCT00729664	Severe sepsis, HIV-infected patients, malignancies	Human IgG4	Bristol-Myers Squibb	Phase I/II
	LY3300054	NCT02791334	Advanced refractory solid tumors	N/A^c^	Eli Lilly	Phase I
	KN035	NCT02827968	Locally advanced or metastatic solid tumors	N/A	3D Medicines (Sichuan, China)	Phase I

^a^Alternative name or prior name of the antibodies are listed in the brackets

^b^NCT number: Clinical trial registry numbers in web of https://clinicaltrials.gov/

^c^N/A, Not Available

Nivolumab displays promising tumor suppressive activity in metastatic melanoma, NSCLC, and metastatic renal cell carcinomas (Brahmer et al., 2010; Topalian et al., 2012). The use of nivolumab has achieved an overall objective response rate (ORR) of 30-40% in multiple clinical trials in patients with melanoma (Topalian et al., 2014; Robert et al., 2015a). Pembrolizumab demonstrates similar efficacy in advanced melanoma. Data from phase III clinical trials on advanced melanoma indicates that patients receiving pembrolizumab show better survival benefits compared to ipilimumab, a MAb targeting CTLA-4 (Robert et al., 2015b). Pembrolizumab is also promising for the treatment of advanced NSCLC (with an ORR of 19%), advanced bladder cancer (with an ORR above 20%), head and neck cancer (with an ORR above 20%), classical Hodgkin’s lymphoma, and triple-negative breast cancer (Garon et al., 2015; Tanguy Y. Seiwert, 2015; Yung-Jue Bang, 2015; Peter H. O’Donnell, 2015).

PD-L1-targeting MAbs are also efficacious in multiple tumors. For instance, atezolizumab (Genentech/Roche) displays promising effects, with an ORR of 43% in PD-L1^+^ patients and an ORR of 11% in PD-L1^-^ patients for the treatment of metastatic urothelial bladder cancer (Powles et al., 2014). In another clinical trial involving NSCLC, melanoma, renal cell carcinoma, *etc*., a response to atezolizumab has more frequently been observed in patients expressing high levels of PD-L1 in tumors, especially when PD-L1 is expressed in TILs (Herbst et al., 2014). Avelumab and durvalumab are also in multiple Phase III clinical trials involving NSCLC, gastric cancer, urothelial cancer, ovarian cancer, *etc*. (Table 1).

However, cases of ineffective PD-1 treatment have also emerged in the observation of clinical trials (Herbst et al., 2014; Tumeh et al., 2014; Rizvi et al., 2015). Considering the complex strategies developed by tumors to evade immune surveillance, pathological types of tumors, mutations of oncogenes and tumor suppressor genes, the stage of disease, and the number of TILs are all essential factors in determining the suitability of immunotherapy. Additionally, the intensity of PD-L1 expression by tumor cells is implicated to be a potential predictor of the efficacy of PD-1 pathway blockade (Topalian et al., 2012).

## STRUCTURAL BASIS OF THE PD-1/PD-L1/L2 RECEPTOR-LIGANDS INTERACTION

**Figure 1:**
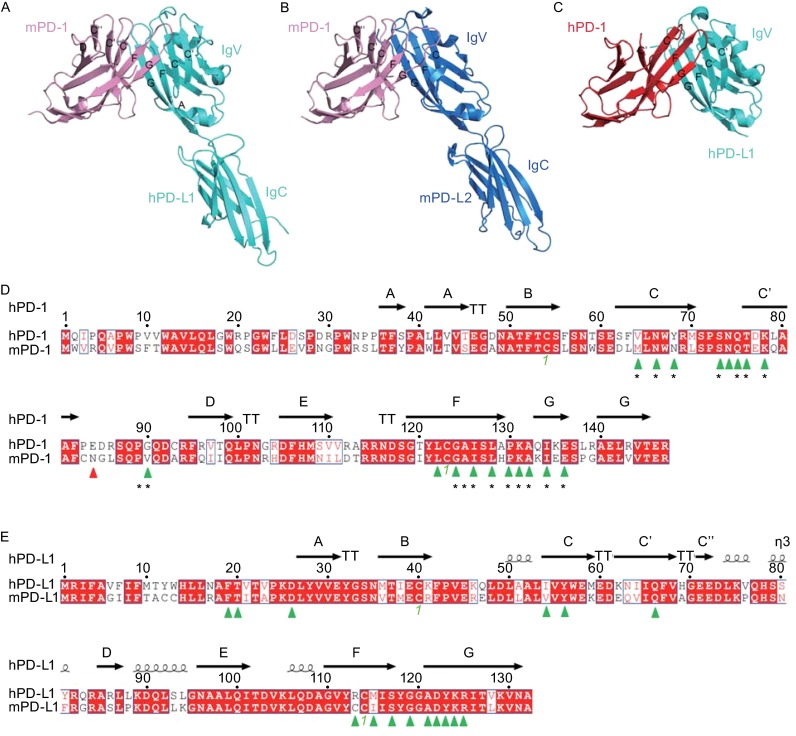
**Overall structure of the mPD-1/hPD-L1, mPD-1/mPD-L2, and hPD-1/hPD-L1 complexes**. Cartoon structures of mPD-1/hPD-L1, mPD-1/mPD-L2, and hPD-1/hPD-L1 complexes. The strands that contribute to interaction are labeled as indicated. A. pink, mPD-1; cyan, hPD-L1. B. pink, mPD-1; sky blue, mPD-L2. C. red, hPD-1; cyan, hPD-L1. D. Sequence alignment of the extracellular IgV domains of hPD-1 and mPD-1. Green triangle labels show the amino acids that interact with both hPD-L1 and mPD-L1 from the complex structures of mPD-1/hPD-L1 and hPD-1/hPD-L1 (PDB: 3BIK, 4ZQK). The red triangle label indicates the amino acids that contribute to the interaction within hPD-1 but not mPD-1. Black asterisks indicate the amino acids within mPD-1 that interact with mPD-L2. E. Sequence alignment of the extracellular IgV domains of hPD-L1 and mPD-L1. Green triangle labels show the amino acids that interact with both hPD-L1 and mPD-L1 from the complex structures of mPD-1/hPD-L1 and hPD-1/hPD-L1 (PDB: 3BIK, 4ZQK). The green number in both D and E indicates the two Cys residues that form an intra-domain disulfide bridge

PD-1 is a type I membrane protein as a member of Ig superfamily with a single extracellular immunoglobulin variable (IgV) domain and is structurally and functionally a monomer (Zhang et al., 2004). On the other hand, its ligands PD-L1 and PD-L2 contain two extracellular Ig domains: the N-terminal IgV domain and C-terminal immunoglobulin constant (IgC) domain (Lazar-Molnar et al., 2008; Lin et al., 2008). The PD-1 extracellular domain adopts an anti-parallel β-sandwich IgV-type monomeric topology, including front sheets (A’ CC’C’’FG) and back sheets (ABED) with a disulfide bridge between Cys54 and Cys123 (Fig.1A–C). Compared to other CD28 family molecules (CTLA-4, CD28, ICOS, *etc*.), PD-1 lacks a Cys in the stalk region, which prevents PD-1 homodimerization (Schwartz et al., 2001). Both monomeric and homodimeric human PD-L1 (hPD-L1) structures were reported by our group and the others, though additional functional evidence is still needed to support these findings (Chen et al., 2010; Zak et al., 2015).

The protein level sequence identity between murine and human PD-1 (mPD-1 and hPD-1) is 64%, while the identity between murine and human PD-L1 (mPD-L1 and hPD-L1) is 77% (Fig. 1D and 1E) (Lin et al., 2008). Cross-species binding has been demonstrated (*i.e.*, mPD-1 can bind to hPD-L1, and hPD-1 can bind to mPD-L1), and the cross-species binding affinities show no significant differences compared to the intra-species interactions (Freeman et al., 2000; Latchman et al., 2001a, b; Zhang et al., 2004; Nomi et al., 2007; Cheng et al., 2013). The amino acids of PD-1 and PD-L1 contributing to the PD-1/PD-L1 interaction are highly conserved between mice and humans, which explains the cross-species binding properties of these paired molecules (Fig. 1D and 1E). However, hPD-1 lacks a well ordered C’’ strand like that found in the IgV fold of mPD-1, which is instead replaced with a flexible loop connecting the C’ and D strands. The flexibility of the C’D loop is supported by the NMR structure and complex structure of pembrolizumab/hPD-1 (discussed below) (Cheng et al., 2013; Na et al., 2016). Additionally, the interaction details of the interface are also quite different between the orthologs (Lin et al., 2008; Zak et al., 2015). Thus, despite the high similarity of the overall structures of human and murine PD-1/PD-L1 and the high conservation of the amino acids involved in the PD-1/PD-L1 interaction between the orthologs, the development and evaluation of hPD-1- or hPD-L1-targeting agents in mouse models deserves more consideration.

Three PD-1/PD-L1/L2 complex structures have so far been determined: mPD-1/hPD-L1, mPD-1/mPD-L2, and hPD-1/hPD-L1 (Lazar-Molnar et al., 2008; Lin et al., 2008; Zak et al., 2015). The interaction of PD-1 and PD-L1 involves both of the front β-sheet faces of their IgV domains (Fig. 1A). The interaction involves the FGCC’C’’ strands, CC’ loop, and FG loop of PD-1 and the AFGCC’ strands of PD-L1 (Fig. 1A and 1C). In comparing the structure of apo-hPD-1 to hPD-1 from hPD-1/hPD-L1 complex structures, significant complex formation-associated conformational changes within hPD-1 are observed involving CC’ loop rearrangement to form hydrogen bonds with hPD-L1 (Zak et al., 2015). In contrast, only minor adjustments of side chains involved in the interaction surface are observed, without significant changes of the backbone, within hPD-L1.

The interaction of mPD-1 with mPD-L2 reveals a similar binding mode to that with PD-L1, which also involves both of the IgV domains with the front β sheet faces interacting with each other (Fig. 1B) (Lazar-Molnar et al., 2008). Most (17/18) of the mPD-1 amino acids that interact with PD-L2 are also involved in the PD-L1 interaction, indicating a similar binding mode of PD-L1 and PD-L2 to PD-1 (Fig. 1D). Thus, agents targeting PD-1 would abrogate the binding of both PD-L1 and PD-L2 to PD-1. However, the detailed interactions of the mPD-1/mPD-L2 interaction significantly differ from that of mPD-1/hPD-L1 (Lazar-Molnar et al., 2008; Lin et al., 2008), suggesting distinct structural basis for the development of PD-L1- and PD-L2-targeting agents.

The reported complex structures reveal the molecular basis of the PD-1/PD-L1/L2 interactions. However, how hPD-1 interacts with hPD-L2 remains undetermined. Moreover, PD-L1 also binds to CD80, which is a ligand of CTLA-4 and CD28, and PD-L2 also has an additional receptor, RGMb. Complex structures of these paired molecules would benefit our understanding of the PD-1/PD-L1/L2 interactions and the development of PD-1/PD-L1/L2 targeting agents in the future.

Based on the complex structure of mPD-1/hPD-L1, Maute et al. have taken advantage of directed evolution of the amino acids in hPD-1 which contributes to the binding with PD-L1 by yeast-surface display to engineer the PD-1 ectodomain as a high-affinity (110 pmol/L) competitive antagonist of PD-L1 (Maute et al., 2015). There are also some peptides, peptidomimetics and small drug-like molecules in preclinical or clinical investigations (Zhan et al., 2016). The recent report on the first nonpeptidic chemical inhibitors that target the PD-1/PD-L1 interaction suggesting that there are “hot spots” on PD-L1 for PD-L1 antagonist drug design (Zak et al., 2016). The structural basis of PD-1 or PD-Ls complexed with these small molecules are also important for drug discovery in the field.

## STRUCTURAL BASIS OF THERAPEUTIC ANTIBODY INTERVENTION

**Figure 2:**
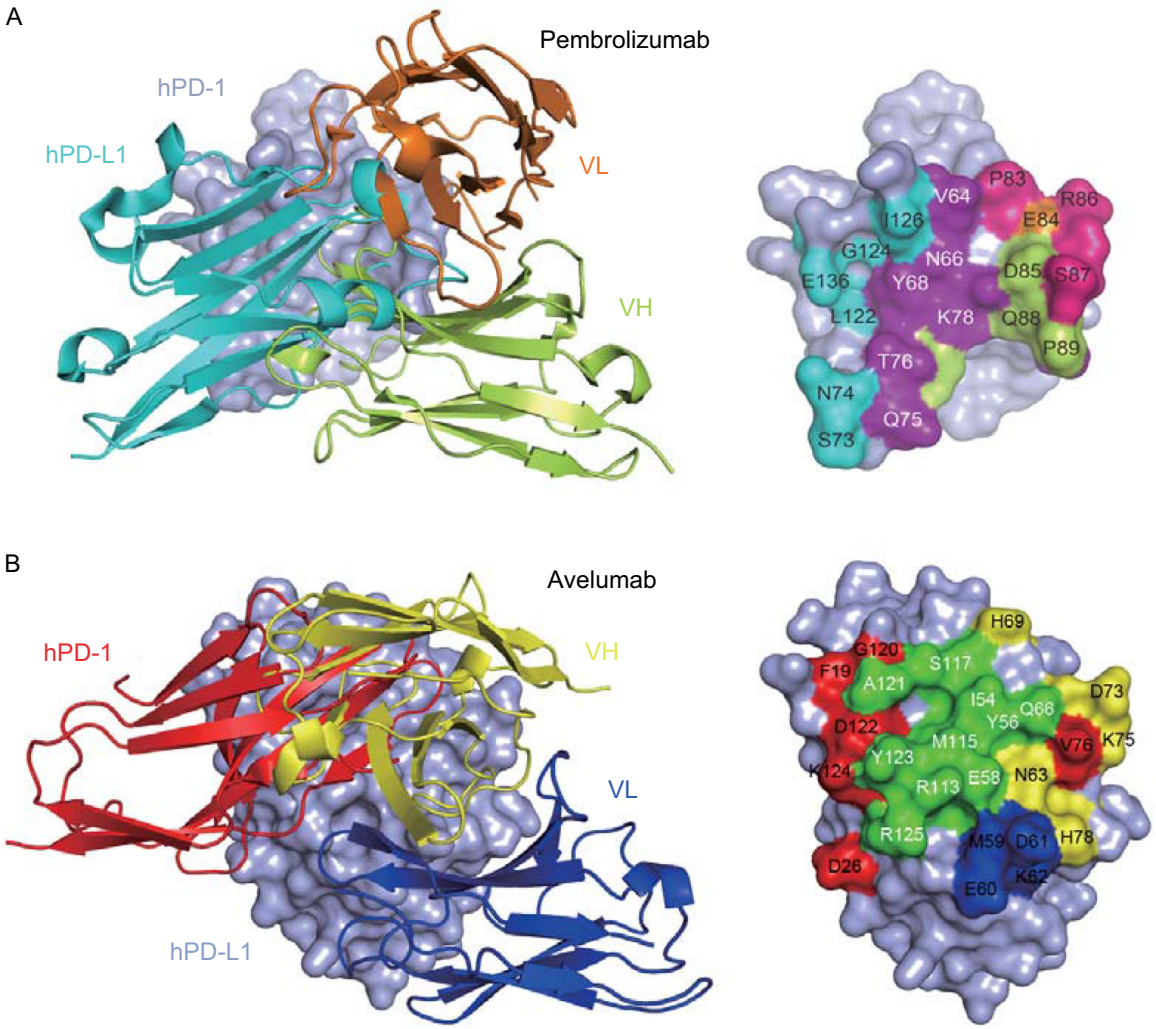
**Structural basis of therapeutic antibody-based PD-1/PD-L1 blockade**. (A) Superimposition of the hPD-1/pembrolizumab-Fab complex structure with the hPD-1/hPD-L1 complex structure. Left, hPD-L1 and pembrolizumab are shown as cartoon (hPD-L1 in cyan, pembrolizumab V_H_ in limon, and V_L_ in orange) while hPD-1 was shown in surface mode. Right, binding surface of hPD-1 for hPD-L1 or pembrolizumab. The binding residues for hPD-L1 on hPD-1 are colored in cyan, whereas residues contacted by the pembrolizumab V_H_ or V_L_ are colored in limon or orange, respectively, and the residues that contacts with both V_H_ and V_L_ are colored in hotpink. The overlapping residues used by both hPD-L1 and pembrolizumab are colored in purple. (B) Superimposition of the hPD-L1/avelumab-scFv complex structure with the hPD-1/hPD-L1 complex structure. Left, hPD-1 and avelumab are shown as cartoon (hPD-1 in red, avelumab-scFv V_H_ in yellow, and V_L_ in blue) while hPD-L1 was shown in surface mode. Right, binding surface of hPD-L1 for hPD-1 or avelumab. The binding residues for hPD-1 on hPD-L1 are colored in red, whereas residues contacted by the avelumab V_H_ or V_L_ are colored in yellow or blue, respectively, and the overlapping residues used by both the receptor hPD-1 and avelumab are colored in green

Crystal structures of the anti-PD-1 pembrolizumab Fab fragment complexed with hPD-1 and the anti-PD-L1 avelumab single chain Fv fragment (scFv) complexed with hPD-L1 have been determined by Na *et al.* (2016) and our group, revealing the molecular basis of therapeutic antibody-based immune checkpoint therapy for tumors (Liu et al., 2016; Na et al., 2016). The interaction of pembrolizumab with hPD-1 is mainly located on two regions: the flexible C’D loop and the C, C’ strands. Unlike the C’’ strand observed in mPD-1, the corresponding region in hPD-1 contains a disordered C’D loop in solution (Fig. 2A left) (Cheng et al., 2013). Though the C’D loop is not involved in the interaction with hPD-L1, it contributes major contacts with pembrolizumab through polar, charged, and hydrophobic contacts. Both the heavy chain (V_H_) and light chain (V_L_) of pembrolizumab are involved in contacting the C’D loop of hPD-1 (Fig. 2A right). The other regions that pembrolizumab interacts with are located on the C and C’ strands of hPD-1, which contribute critical contacts with hPD-L1 (Fig. 2A right). Thus, the blockade of the hPD-1/hPD-L1 interaction by pembrolizumab occurs predominantly by binding to the C’D loop and overlaps binding to the C and C’ strands to compete with the binding of hPD-L1.

Structural analysis of the interaction of avelumab with hPD-1 reveals that avelumab utilizes both V_H_ and V_L_ to bind to the IgV domain of PD-L1 on its side (Liu et al., 2016). The V_H_ of avelumab dominates the binding to hPD-L1 by all three complementarity determining regions (CDR) loops, while V_L_ contributes partial contacts by the CDR1 and CDR3 loops, leaving V_L_ CDR2 without any binding to hPD-L1 (Fig. 2B left). The binding epitope region of avelumab on hPD-L1 predominantly consists of the C, C’, F, and G strands and the CC’ loop of hPD-L1. The blockade binding of avelumab is mainly occupied by the V_H_ chain, with minor contribution from V_L_ chain (Fig. 2B right). The detailed analysis of the buried surface on hPD-L1 reveals that the overlapping area of avelumab and hPD-1 is mainly located on the F and G strands, which are predominantly occupied by the HCDR2 loop of avelumab (Fig. 2B right). Therefore, the mechanism of avelumab blockade involves the protruding HCDR2 loop dominating the hPD1 binding region and competing for the binding of hPD-1 to hPD-L1.

The binding affinities (*K*_*d*_) of pembrolizumab to hPD-1 and avelumab to hPD-L1 are 27.0 pmol/L and 42.1 pmol/L, respectively (Na et al., 2016). On the other hand, the binding affinity between hPD-1 and hPD-L1 is 0.77–8.2 μmol/L (Collins et al., 2002; Butte et al., 2007; Cheng et al., 2013), which is much weaker than that of the antibodies. The strong binding of pembrolizumab to hPD-1 and avelumab to hPD-L1 would enable the binding priority of the therapeutic antibodies with checkpoint molecules and subsequent blockade of the hPD-1/hPD-L1 interaction.

There are yet more therapeutic antibodies targeting PD-1/PD-L1/L2 in clinical use or clinical trials (*e.g.*, nivolumab, atezolizumab, and durvalumab). Whether these antibodies utilize the same blockade mode as pembrolizumab or avelumab remains undetermined. Moreover, whether there are hot-spots on PD-1 or PD-L1 to be targeted by different therapeutic antibodies requires further investigation. All of these findings would benefit the development of therapeutic agents targeting the PD-1 pathway to disrupt the PD-1/PD-L1 interaction.

## CONCLUSION AND PERSPECTIVES

The success of checkpoint blockade therapy has brought immunotherapy from the corner to center stage in fighting against human cancers, especially for solid tumors. In contrast to other strategies that prime or boost cancer-specific immune responses, immune checkpoint blockade therapy targets tumor-induced immune defects and revives existing tumor-specific T cells to kill tumor cells. The PD-1/PD-L1 pathway has been taking the priority that single use of PD-1 or PD-L1 blockade antibodies can eliminate tumors in at least a portion of patients. Though clinical success with anti-PD therapy has been achieved, the molecular basis of the PD-1/PD-L1/L2 interaction and PD-L1/L2 interaction with other receptors needs to be further investigated. The recently reported therapeutic antibody complex structures with PD-1 or PD-L1 make it clear how the therapeutic antibodies work, providing a new approach to modify these antibodies for the better effects. However, more antibody/PD-1 (or PD-L1, PD-L2) interaction details are still needed to define the antibody targeting hot-spots and to better design PD-1/PD-L1/L2 antagonists for tumor treatment. Such efforts will pave a way to improve the efficacy of antibody targeting the PD-1 pathway and prolong survival in advanced cancer patients.

## References

[CR1] Agata Y, Kawasaki A, Nishimura H, Ishida Y, Tsubata T, Yagita H, Honjo T (1996). Expression of the PD-1 antigen on the surface of stimulated mouse T and B lymphocytes. Int Immunol.

[CR2] Ahmadzadeh M, Johnson LA, Heemskerk B, Wunderlich JR, Dudley ME, White DE, Rosenberg SA (2009). Tumor antigen-specific CD8 T cells infiltrating the tumor express high levels of PD-1 and are functionally impaired. Blood.

[CR3] Bang YJ, Chung HC, Shankaran V, Geva R, Catenacci DVT, Gupta S, Eder JP, Berger R, Gonzalez EJ, Ray A, Dolled-Filhart M, Emancipator K, Pathiraja K, Lunceford JK, Cheng JD, Koshiji J, Muro K (2015) Relationship between PD-L1 expression and clinical outcomes in patients with advanced gastric cancer treated with the anti-PD-1 monoclonal antibody pembrolizumab (MK-3475) in KEYNOTE-012. J Clin Oncol 33

[CR4] Barber DL, Wherry EJ, Masopust D, Zhu B, Allison JP, Sharpe AH, Freeman GJ, Ahmed R (2006). “Exhausted” T cells: good or bad depends on your point of view - Restoring function in exhausted CD8 T cells during chronic viral infection. Liver Transpl.

[CR5] Barber DL, Wherry EJ, Masopust D, Zhu BG, Allison JP, Sharpe AH, Freeman GJ, Ahmed R (2006). Restoring function in exhausted CD8 T cells during chronic viral infection. Nature.

[CR6] Benvenuti F, Lagaudriere-Gesbert C, Grandjean I, Jancic C, Hivroz C, Trautmann A, Lantz O, Amigorena S (2004). Dendritic cell maturation controls adhesion, synapse formation, and the duration of the interactions with naive T lymphocytes. J Immunol.

[CR7] Blackburn SD, Shin H, Haining WN, Zou T, Workman CJ, Polley A, Betts MR, Freeman GJ, Vignali DAA, Wherry EJ (2009). Coregulation of CD8(+) T cell exhaustion by multiple inhibitory receptors during chronic viral infection. Nat Immunol.

[CR8] Blank C, Kuball J, Voelkl S, Wiendl H, Becker B, Walter B, Majdic O, Gajewski TF, Theobald M, Andreesen R (2006). Blockade of PD-L1 (B7-H1) augments human tumor-specific T cell responses in vitro. Int J Cancer.

[CR9] Brahmer JR, Drake CG, Wollner I, Powderly JD, Picus J, Sharfman WH, Stankevich E, Pons A, Salay TM, McMiller TL (2010). Phase I study of single-agent anti-programmed death-1 (MDX-1106) in refractory solid tumors: safety, clinical activity, pharmacodynamics, and immunologic correlates. J Clin Oncol.

[CR10] Bretscher P, Cohn M (1970). A theory of self-nonself discrimination. Science.

[CR11] Brown JA, Dorfman DM, Ma FR, Sullivan EL, Munoz O, Wood CR, Greenfield EA, Freeman GJ (2003). Blockade of programmed death-1 Ligands on dendritic cells enhances T cell activation and cytokine production. J Immunol.

[CR12] Butte MJ, Keir ME, Phamduy TB, Sharpe AH, Freeman GJ (2007). Programmed death-1 ligand 1 interacts specifically with the B7-1 costimulatory molecule to inhibit T cell responses. Immunity.

[CR13] Callahan MK, Postow MA, Wolchok JD (2016). Targeting T Cell co-receptors for cancer therapy. Immunity.

[CR14] Chang WS, Kim JY, Kim YJ, Kim YS, Lee JM, Azuma M, Yagita H, Kang CY (2008). Cutting edge: programmed death-1/programmed death ligand 1 interaction regulates the induction and maintenance of invariant NKT cell anergy. J Immunol.

[CR15] Chapman PB, D’Angelo SP, Wolchok JD (2015). Rapid eradication of a bulky melanoma mass with one dose of immunotherapy. N Eng J Med.

[CR16] Chatterjee P, Patsoukis N, Freeman GJ, Boussiotis VA (2013). Distinct roles of PD-1 Itsm and ITIM In regulating interactions with SHP-2, ZAP-70 and Lck, and PD-1-mediated inhibitory function. Blood.

[CR17] Chemnitz JM, Parry RV, Nichols KE, June CH, Riley JL (2004). SHP-1 and SHP-2 associate with immunoreceptor tyrosine-based switch motif of programmed death 1 upon primary human T cell stimulation, but only receptor ligation prevents T cell activation. J Immunol.

[CR18] Chemnitz JM, Eggle D, Driesen J, Classen S, Riley JL, Debey-Pascher S, Beyer M, Popov A, Zander T, Schultze JL (2007). RNA fingerprints provide direct evidence for the inhibitory role of TGF beta and PD-1 on CD4(+) T cells in Hodgkin lymphoma. Blood.

[CR19] Chen L, Han X (2015). Anti-PD-1/PD-L1 therapy of human cancer: past, present, and future. J Clin Invest.

[CR20] Chen Y, Liu P, Gao F, Cheng H, Qi J, Gao GF (2010). A dimeric structure of PD-L1: functional units or evolutionary relics?. Protein Cell.

[CR21] Cheng X, Veverka V, Radhakrishnan A, Waters LC, Muskett FW, Morgan SH, Huo J, Yu C, Evans EJ, Leslie AJ (2013). Structure and interactions of the human programmed cell death 1 receptor. J Biol Chem.

[CR22] Collins AV, Brodie DW, Gilbert RJ, Iaboni A, Manso-Sancho R, Walse B, Stuart DI, van der Merwe PA, Davis SJ (2002). The interaction properties of costimulatory molecules revisited. Immunity.

[CR23] Cunningham AJ, Lafferty KJ (1977). A simple conservative explanation of the H-2 restriction of interactions between lymphocytes. Scand J Immunol.

[CR24] Curiel TJ, Wei S, Dong HD, Alvarez X, Cheng P, Mottram P, Krzysiek R, Knutson KL, Daniel B, Zimmermann MC (2003). Blockade of B7-H1 improves myeloid dendritic cell-mediated antitumor immunity. Nat Med.

[CR25] Dong HD, Strome SE, Salomao DR, Tamura H, Hirano F, Flies DB, Roche PC, Lu J, Zhu GF, Tamada K (2002). Tumor-associated B7-H1 promotes T-cell apoptosis: a potential mechanism of immune evasion. Nat Med.

[CR26] Francisco LM, Salinas VH, Brown KE, Vanguri VK, Freeman GJ, Kuchroo VK, Sharpe AH (2009). PD-L1 regulates the development, maintenance, and function of induced regulatory T cells. J Exp Med.

[CR27] Freeman GJ, Long AJ, Iwai Y, Bourque K, Chernova T, Nishimura H, Fitz LJ, Malenkovich N, Okazaki T, Byrne MC (2000). Engagement of the PD-1 immunoinhibitory receptor by a novel B7 family member leads to negative regulation of lymphocyte activation. J Exp Med.

[CR28] Gandini S, Massi D, Mandala M (2016). PD-L1 expression in cancer patients receiving anti PD-1/PD-L1 antibodies: A systematic review and meta-analysis. Crit Rev Oncol Hematol.

[CR29] Gao GF, Jakobsen BK (2000). Molecular interactions of coreceptor CD8 and MHC class I: the molecular basis for functional coordination with the T-cell receptor. Immunol Today.

[CR30] Gao GF, Rao Z, Bell JI (2002). Molecular coordination of alphabeta T-cell receptors and coreceptors CD8 and CD4 in their recognition of peptide-MHC ligands. Trends Immunol.

[CR31] Garon EB, Rizvi NA, Hui R, Leighl N, Balmanoukian AS, Eder JP, Patnaik A, Aggarwal C, Gubens M, Horn L (2015). Pembrolizumab for the treatment of non-small-cell lung cancer. N Engl J Med.

[CR32] Ghebeh H, Mohammed S, Al-Omair A, Qattan A, Lehe C, Al-Qudaihi G, Elkum N, Alshabanah M, Bin Amer S, Tulbah A (2006). The B7-H1 (PD-L1) T lymphocyte-inhibitory molecule is expressed in breast cancer patients with infiltrating ductal carcinoma: Correlation with important high-risk prognostic factors. Neoplasia.

[CR33] Hamanishi J, Mandai M, Iwasaki M, Okazaki T, Tanaka Y, Yamaguchi K, Higuchi T, Yagi H, Takakura K, Minato N (2007). Programmed cell death 1 ligand 1 and tumor-infiltrating CD8(+) T lymphocytes are prognostic factors of human ovarian cancer. Proc Natl Acad Sci U S A.

[CR34] Herbst RS, Soria JC, Kowanetz M, Fine GD, Hamid O, Gordon MS, Sosman JA, McDermott DF, Powderly JD, Gettinger SN (2014). Predictive correlates of response to the anti-PD-L1 antibody MPDL3280A in cancer patients. Nature.

[CR35] Hino R, Kabashima K, Kato Y, Yagi H, Nakamura M, Honjo T, Okazaki T, Tokura Y (2010). Tumor cell expression of programmed Cell Death-1 Ligand 1 Is a prognostic factor for malignant melanoma. Cancer.

[CR36] Hirano F, Kaneko K, Tamura H, Dong HD, Wang SD, Ichikawa M, Rietz C, Flies DB, Lau JS, Zhu GF (2005). Blockade of B7-H1 and PD-1 by monoclonal antibodies potentiates cancer therapeutic immunity. Cancer Res.

[CR37] Ishida Y, Agata Y, Shibahara K, Honjo T (1992). Induced expression of Pd-1, a novel member of the immunoglobulin gene superfamily, upon programmed cell-death. EMBO J.

[CR38] Iwai Y, Ishida M, Tanaka Y, Okazaki T, Honjo T, Minato N (2002). Involvement of PD-L1 on tumor cells in the escape from host immune system and tumor immunotherapy by PD-L1 blockade. Proc Natl Acad Sci U S A.

[CR39] John LB, Devaud C, Duong CPM, Yong CS, Beavis PA, Haynes NM, Chow MT, Smyth MJ, Kershaw MH, Darcy PK (2013). Anti-PD-1 antibody therapy potently enhances the eradication of established tumors by gene-modified T cells. Clin Cancer Res.

[CR40] Kataoka K, Shiraishi Y, Takeda Y, Sakata S, Matsumoto M, Nagano S, Maeda T, Nagata Y, Kitanaka A, Mizuno S (2016). Aberrant PD-L1 expression through 3 ‘-UTR disruption in multiple cancers. Nature.

[CR41] Kinter AL, Godbout EJ, McNally JP, Sereti I, Roby GA, O’Shea MA, Fauci AS (2008). The common gamma-Chain Cytokines IL-2, IL-7, IL-15, and IL-21 induce the expression of programmed Death-1 and its ligands. J Immunol.

[CR42] Kleffel S, Posch C, Barthel SR, Mueller H, Schlapbach C, Guenova E, Elco CP, Lee N, Juneja VR, Zhan Q (2015). Melanoma cell-intrinsic PD-1 receptor functions promote tumor growth. Cell.

[CR43] Lafferty KJ, Cunningham AJ (1975). A new analysis of allogeneic interactions. Aust J Exp Biol Med Sci.

[CR44] Latchman Y, Wood C, Chernova T, Chaudhary D, Borde M, Chernova I, Iwai Y, Long AJ, Brown JA, Nunes R, Greenfield EA, Bourque K, Boussiotis VA, Carter LL, Carreno BM, Malenkovich N, Nishimura H, Okazaki T, Honjo T, Sharpe AH, Freeman GJ (2001). PD-L2 is a second ligand for PD-1 and inhibits T cell activation. Nat Immunol.

[CR45] Latchman Y, Wood C, Chemova T, Iwai Y, Malenkovich N, Long A, Bourque K, Boussiotis V, Nishimura H, Honjo T (2001). PD-L2, a novel B7 homologue, is a second ligand for PD-1 and inhibits T cell activation. Faseb J.

[CR46] Lazar-Molnar E, Yan Q, Cao E, Ramagopal U, Nathenson SG, Almo SC (2008). Crystal structure of the complex between programmed death-1 (PD-1) and its ligand PD-L2. Proc Natl Acad Sci U S A.

[CR47] Lesterhuis WJ, Steer H, Lake RA (2011). PD-L2 is predominantly expressed by Th2 cells. Mol Immunol.

[CR48] Li Y, Li F, Jiang F, Lv X, Zhang R, Lu A, Zhang G (2016). A mini-review for cancer immunotherapy: molecular understanding of PD-1/PD-L1 pathway & translational blockade of immune checkpoints. Int J Mol Sci.

[CR49] Lin DY, Tanaka Y, Iwasaki M, Gittis AG, Su HP, Mikami B, Okazaki T, Honjo T, Minato N, Garboczi DN (2008). The PD-1/PD-L1 complex resembles the antigen-binding Fv domains of antibodies and T cell receptors. Proc Natl Acad Sci U S A.

[CR50] Liu JZ, Hamrouni A, Wolowiec D, Coiteux V, Kuliczkowski K, Hetuin D, Saudemont A, Quesnel B (2007). Plasma cells from multiple myeloma patients express B7-H1 (PD-L1) and increase expression after stimulation with IFN-gamma and TLR ligands via a MyD88-, TRAF6-, and MEK-dependent pathway. Blood.

[CR51] Liu Y, Yu YY, Yang SG, Zeng B, Zhang ZH, Jiao GH, Zhang Y, Cai LM, Yang RC (2009). Regulation of arginase I activity and expression by both PD-1 and CTLA-4 on the myeloid-derived suppressor cells. Cancer Immunol Immunother.

[CR52] Liu K, Tan S, Chai Y, Chen D, Song H, Zhang CW, Shi Y, Liu J, Tan W, Lyu J, Gao S, Yan J, Qi J, Gao GF (2016). Structural basis of anti-PD-L1 monoclonal antibody avelumab for tumor therapy. Cell Res.

[CR53] Mahoney KM, Rennert PD, Freeman GJ (2015). Combination cancer immunotherapy and new immunomodulatory targets. Nat Rev Drug Disc.

[CR54] Maute RL, Gordon SR, Mayer AT, McCracken MN, Natarajan A, Ring NG, Kimura R, Tsai JM, Manglik A, Kruse AC (2015). Engineering high-affinity PD-1 variants for optimized immunotherapy and immuno-PET imaging. Proc Natl Acad Sci U S A.

[CR55] Na Z, Yeo SP, Bharath SR, Bowler MW, Balikci E, Wang CI, Song H (2016). Structural basis for blocking PD-1-mediated immune suppression by therapeutic antibody pembrolizumab. Cell Res.

[CR56] Nishimura H, Agata Y, Kawasaki A, Sato M, Imamura S, Minato N, Yagita H, Nakano T, Honjo T (1996). Developmentally regulated expression of the PD-1 protein on the surface of double-negative (CD4(-)CD8(-)) thymocytes. Int Immunol.

[CR57] Nishimura H, Nose M, Hiai H, Minato N, Honjo T (1999). Development of lupus-like autoimmune diseases by disruption of the PD-1 gene encoding an ITIM motif-carrying immunoreceptor. Immunity.

[CR58] Nomi T, Sho M, Akahori T, Hamada K, Kubo A, Kanehiro H, Nakamura S, Enomoto K, Yagita H, Azuma M (2007). Clinical significance and therapeutic potential of the programmed death-1 ligand/programmed death-1 pathway in human pancreatic cancer. Clin Cancer Res.

[CR59] O’Donnell PH, Pilmack ER, Bellmunt J, Berger R, Montgomery RB, Heath K, Dolled-Filhart M, Pathiraja K, Gause CK, Cheng JD, Perini RF, Gupta S (2015). Pembrolizumab (Pembro; MK-3475) for advanced urothelial cancer: Results of a phase IB study. J Clin Oncol 33.

[CR60] Ohigashi Y, Sho M, Yamada Y, Tsurui Y, Hamada K, Ikeda N, Mizuno T, Yoriki R, Kashizuka H, Yane K (2005). Clinical significance of programmed death-1 ligand-1 and programmed death-1 ligand-2 expression in human esophageal cancer. Clin Cancer Res.

[CR61] Okazaki T, Maeda A, Nishimura H, Kurosaki T, Honjo T (2001). PD-1 immunoreceptor inhibits B cell receptor-mediated signaling by recruiting src homology 2-domain-containing tyrosine phosphatase 2 to phosphotyrosine. Proc Natl Acad Sci U S A.

[CR62] Palucka AK, Coussens LM (2016). The basis of oncoimmunology. Cell.

[CR63] Petrovas C, Casazza JP, Brenchley JM, Price DA, Gostick E, Adams WC, Precopio ML, Schacker T, Roederer M, Douek DC (2006). PD-1 is a regulator of virus-specific CD8(+) T cell survival in HIV infection. J Exp Med.

[CR64] Postow MA, Chesney J, Pavlick AC, Robert C, Grossmann K, McDermott D, Linette GP, Meyer N, Giguere JK, Agarwala SS (2015). Nivolumab and ipilimumab versus ipilimumab in untreated melanoma. N Engl J Med.

[CR65] Powles T, Eder JP, Fine GD, Braiteh FS, Loriot Y, Cruz C, Bellmunt J, Burris HA, Petrylak DP, Teng SL (2014). MPDL3280A (anti-PD-L1) treatment leads to clinical activity in metastatic bladder cancer. Nature.

[CR66] Rizvi NA, Hellmann MD, Snyder A, Kvistborg P, Makarov V, Havel JJ, Lee W, Yuan J, Wong P, Ho TS (2015). Cancer immunology. Mutational landscape determines sensitivity to PD-1 blockade in non-small cell lung cancer. Science.

[CR67] Robert C, Long GV, Brady B, Dutriaux C, Maio M, Mortier L, Hassel JC, Rutkowski P, McNeil C, Kalinka-Warzocha E (2015). Nivolumab in previously untreated melanoma without BRAF mutation. N Engl J Med.

[CR68] Robert C, Schachter J, Long GV, Arance A, Grob JJ, Mortier L, Daud A, Carlino MS, McNeil C, Lotem M (2015). Pembrolizumab versus ipilimumab in advanced melanoma. N Engl J Med.

[CR69] Schildberg FA, Klein SR, Freeman GJ, Sharpe AH (2016). Coinhibitory pathways in the B7-CD28 ligand-receptor family. Immunity.

[CR70] Schwartz JC, Zhang X, Fedorov AA, Nathenson SG, Almo SC (2001). Structural basis for co-stimulation by the human CTLA-4/B7-2 complex. Nature.

[CR71] Sheppard KA, Fitz LJ, Lee JM, Benander C, George JA, Wooters J, Qiu YC, Jussif JM, Carter LL, Wood CR (2004). PD-1 inhibits T-cell receptor induced phosphorylation of the ZAP70/CD3 zeta signalosome and downstream signaling to PKC theta. Febs Lett.

[CR72] Shimauchi T, Kabashima K, Nakashima D, Sugita K, Yamada Y, Hino R, Tokura Y (2007). Augmented expression of programmed death-1 in both neoplastic and non-neoplastic CD4(+) T-cells in adult T-cell leukemia/lymphoma. Int J Cancer.

[CR73] Tan S, Gao GF (2015). New hope for cancer treatment: Cancer Immunotherapy. Chin Sci Bull.

[CR74] Tanguy Y. Seiwert, B.B., Jared Weiss, Joseph Paul Eder, Jennifer Yearley, Erin Murphy, Michael Nebozhyn, Terri McClanahan, Mark Ayers, Jared K. Lunceford, Ranee Mehra, Karl Heath, Jonathan D. Cheng and Laura Q. Chow (2015). Inflamed-phenotype gene expression signatures to predict benefit from the anti-PD-1 antibody pembrolizumab in PD-L1+ head and neck cancer patients. J Clin Oncol 33.

[CR75] Thompson RH, Gillett MD, Cheville JC, Lohse CM, Dong HD, Webster WS, Krejci KG, Lobo JR, Sengupta S, Chen LP (2005). Costimulatory B7-H1 in renal cell carcinoma patients: Indicator of tumor aggressiveness and potential therapeutic target. J Urol.

[CR76] Topalian SL, Hodi FS, Brahmer JR, Gettinger SN, Smith DC, McDermott DF, Powderly JD, Carvajal RD, Sosman JA, Atkins MB (2012). Safety, activity, and immune correlates of anti-PD-1 antibody in cancer. N Engl J Med.

[CR77] Topalian SL, Sznol M, McDermott DF, Kluger HM, Carvajal RD, Sharfman WH, Brahmer JR, Lawrence DP, Atkins MB, Powderly JD (2014). Survival, durable tumor remission, and long-term safety in patients with advanced melanoma receiving nivolumab. J Clin Oncol.

[CR78] Tseng SY, Otsuji M, Gorski K, Huang X, Slansky JE, Pai SI, Shalabi A, Shin T, Pardoll DM, Tsuchiya H (2001). B7-DC, a new dendritic cell molecule with potent costimulatory properties for T cells. J Exp Med.

[CR79] Tumeh PC, Harview CL, Yearley JH, Shintaku IP, Taylor EJM, Robert L, Chmielowski B, Spasic M, Henry G, Ciobanu V (2014). PD-1 blockade induces responses by inhibiting adaptive immune resistance. Nature.

[CR80] Wang HY, Lee DA, Peng G, Guo Z, Li Y, Kiniwa Y, Shevach EM, Wang RF (2004). Tumor-specific human CD4+ regulatory T cells and their ligands: implications for immunotherapy. Immunity.

[CR81] Wherry EJ, Blattman JN, Murali-Krishna K, van der Most R, Ahmed R (2003). Viral persistence alters CD8 T-cell immunodominance and tissue distribution and results in distinct stages of functional impairment. J Virol.

[CR82] Wu CP, Zhu YB, Jiang JT, Zhao JM, Zhang XG, Xu N (2006). Immunohistochemical localization of programmed death-1 ligand-1 (PD-L1) in gastric carcinoma and its clinical significance. Acta Histochem.

[CR83] Xiao Y, Yu S, Zhu B, Bedoret D, Bu X, Francisco LM, Hua P, Duke-Cohan JS, Umetsu DT, Sharpe AH (2014). RGMb is a novel binding partner for PD-L2 and its engagement with PD-L2 promotes respiratory tolerance. J Exp Med.

[CR84] Yamazaki T, Akiba H, Iwai H, Matsuda H, Aoki M, Tanno Y, Shin T, Tsuchiya H, Pardoll DM, Okumura K (2002). Expression of programmed death 1 ligands by murine T cells and APC. J Immunol.

[CR85] Zak KM, Kitel R, Przetocka S, Golik P, Guzik K, Musielak B, Domling A, Dubin G, Holak TA (2015). Structure of the Complex of Human Programmed Death 1, PD-1, and Its Ligand PD-L1. Structure.

[CR86] Zak KM, Grudnik P, Guzik K, Zieba BJ, Musielak B, Domling A, Dubin G, Holak TA (2016). Structural basis for small molecule targeting of the programmed death ligand 1 (PD-L1). Oncotarget.

[CR87] Zhan MM, Hu XQ, Liu XX, Ruan BF, Xu J, Liao C (2016). From monoclonal antibodies to small molecules: the development of inhibitors targeting the PD-1/PD-L1 pathway. Drug Discov Today.

[CR88] Zhang X, Schwartz JC, Guo X, Bhatia S, Cao E, Lorenz M, Cammer M, Chen L, Zhang ZY, Edidin MA (2004). Structural and functional analysis of the costimulatory receptor programmed death-1. Immunity.

[CR89] Zhang B, Chikuma S, Hori S, Fagarasan S, Honjo T (2016). Nonoverlapping roles of PD-1 and FoxP3 in maintaining immune tolerance in a novel autoimmune pancreatitis mouse model. Proc Natl Acad Sci U S A.

[CR90] Zou W, Wolchok JD, Chen L (2016). PD-L1 (B7-H1) and PD-1 pathway blockade for cancer therapy: mechanisms, response biomarkers, and combinations. Sci Transl Med.

